# Responses of bacterial community structure and denitrifying bacteria in biofilm to submerged macrophytes and nitrate

**DOI:** 10.1038/srep36178

**Published:** 2016-10-26

**Authors:** Songhe Zhang, Si Pang, Peifang Wang, Chao Wang, Chuan Guo, Felix Gyawu Addo, Yi Li

**Affiliations:** 1 Ministry of Education Key Laboratory of Integrated Regulation and Resource Development on Shallow Lakes, College of Environment, Hohai University, Nanjing 210098, China

## Abstract

Submerged macrophytes play important roles in constructed wetlands and natural water bodies, as these organisms remove nutrients and provide large surfaces for biofilms, which are beneficial for nitrogen removal, particularly from submerged macrophyte-dominated water columns. However, information on the responses of biofilms to submerged macrophytes and nitrogen molecules is limited. In the present study, bacterial community structure and denitrifiers were investigated in biofilms on the leaves of four submerged macrophytes and artificial plants exposed to two nitrate concentrations. The biofilm cells were evenly distributed on artificial plants but appeared in microcolonies on the surfaces of submerged macrophytes. Proteobacteria was the most abundant phylum in all samples, accounting for 27.3–64.8% of the high-quality bacterial reads, followed by Chloroflexi (3.7–25.4%), Firmicutes (3.0–20.1%), Acidobacteria (2.7–15.7%), Actinobacteria (2.2–8.7%), Bacteroidetes (0.5–9.7%), and Verrucomicrobia (2.4–5.2%). Cluster analysis showed that bacterial community structure can be significantly different on macrophytes versus from those on artificial plants. Redundancy analysis showed that electrical conductivity and nitrate concentration were positively correlated with Shannon index and operational taxonomic unit (OTU) richness (log_10_ transformed) but somewhat negatively correlated with microbial density. The relative abundances of five denitrifying genes were positively correlated with nitrate concentration and electrical conductivity but negatively correlated with dissolved oxygen.

Aquatic macrophytes play an important role in water environments and have been used in constructed wetlands, particularly in surface-flow wetlands, which are the most similar to natural environments, providing permanent standing waters and conditions favorable for wetland plant species^1^,^2^. The plant systems in wetlands play key roles in the removal of nitrogen, one of the primary pollutants leading to eutrophication in aquatic environments and subsequent toxicity to aquatic organisms. On the one hand, wetland plants can supply denitrifying bacteria with organic carbon and suitable attachment surfaces, while on the other hand, litter accumulation and decomposition promote the development of anaerobic conditions, which favor denitrification^3^,^4^,^5^. However, the dynamics of the microbial community structure and its relationship to nitrogen removal processes and operational parameters in wetlands are far from clear, as available species-specific data concerning microbial communities in different types of wetlands are scarce^1^.

Nitrogen can be removed from wetlands through physical, chemical, and biological processes^6^. Biological denitrification is the major pathway for nitrogen removal in most wetlands through the reduction of nitrate or nitrite to gases (nitrous oxide or dinitrogen)^7^, as nitrogen stored in sediment systems and macrophytes, algae and microorganisms might subsequently be released again^8^. Denitrification uses nitrogen compounds as alternative electron acceptors for energy production^9^. Denitrifiers carrying metalloenzymes, including nitrate reductase, nitrite reductase, nitric oxide reductase and nitrous oxide reductase, are involved in the reduction of nitrate to dinitrogen gas. The genes encoding the catalytic subunit of denitrifying reductases, e.g., *nar*G, *nap*A, *nir*S, *nir*K, *nor*B and *nos*Z, have been detected in the genomes of numerous groups of bacteria and archaea^10^ and have been used to monitor denitrifier populations in various environments^9^,^11^,^12^.

Denitrification is achieved under anaerobic or anoxic conditions^13^,^14^. However, sporadic reports on aerobic denitrifiers have induced aerobic denitrification using nitrate-oxidizing agents under aerobic conditions^9^. Aerobic denitrification provides a new pathway for the biological modulation of nitrogen. In wetlands, the roots of emergent macrophytes, widely planted in conveniently operated and managed wetlands, can secrete oxygen, generating an aerobic micro-environment^15^. Growing in the deep sections of wetlands, submerged macrophytes can provide a large area for attached biofilm (epiphytic microbes) and provide oxygen for nitrifiers in biofilms, surficial sediments or daytime water columns^8^. At night, respiratory consumption in the dense stands of submerged vegetation regions might result in a shift from aerobic to anaerobic bacterial respiration, which is beneficial for denitrification^16^.

Submerged macrophyte-biofilm systems play an important role in nitrification and denitrification^16^. Bourgues and Hart^17^ showed that the potential denitrification activity of the biofilm on dominant macrophytes was comparable with the activities measured in the sediments, and biofilms play a key role in removing nitrogen loads. However, there is no direct evidence confirming the occurrence of aerobic denitrification in complex wetland systems. In addition, the presence and types of macrophytes affect denitrification rates^18^. Therefore, bacterial community structure and denitrifier communities must be examined to assess their responses to plant species and high nitrate concentrations.

The aim of the present study was to fill the knowledge gap concerning the underlying bacterial community structure and denitrification genes in biofilms attached to five substrates (four submerged macrophytes and an artificial plant) at two nitrate concentrations. We explored the following objectives to elucidate the shifts of bacterial (and archaeal) community and denitrifiers in biofilm under the impact of substrates and nitrate levels: (i) to assess and compare the bacterial communities in biofilm attached to five substrates under two nitrate concentrations, (ii) to determine the five functional gene groups in all biofilm samples underlying their responses to nitrate, and (iii) to analyze the responses of biofilm parameters and denitrification genes in biofilms to environmental parameters. The results of the present study will have important implications for understanding the role of biofilms in wetlands and provide useful information for modifying the design of these areas.

## Results

### Microbes in biofilms

**Figure 1:**
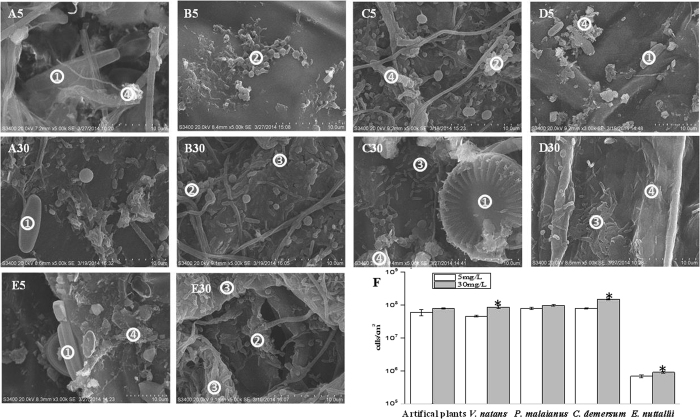
SEM imaging of the biofilms (A5-E5, 5 mg L^−1^ NO_3_^_^N; A30-E30, 30 mg L^−1^ NO_3_^_^N) and microbial densities (**F**) on five substrates (**A**), artificial plants; (**B**) *E*. *nuttallii*; (**C**) *P*. *malaianus*; (**D**) *V*. *natans;* (**E**) *C*. *demersum*). (1) Algae; (2) cocci aggregates; (3) bacillus aggregates; and (4) organic matter or extracellular polymers. *Indicates significant differences at p < 0.05.

Scanning electron microscopy (SEM) was employed to investigate the morphology of biofilms attached to the leaf surfaces of four submerged macrophytes (*Vallisneria natans*, VN; *Potamogeton malaianus*, PM; *Ceratophyllum demersum* CD; and *Elodea nuttallii*, EN) and artificial plants exposed to two levels of nitrate (Figs 1 and S2). The biofilms were not evenly distributed on the surface of the four bio-substrates (Figure S2) but almost covered the entire surface of artificial plants. The surface of the substrates adsorbed a large number of particles, including inorganic particles, bacteria (mainly cocci, *Bacillus* and other forms of bacteria) and algae. After DAPI staining, the microbial densities in the biofilm were investigated using fluorescence microscopy and ranged from 10^5^ to 10^7^ cells cm^−2^ (Fig. 1F). The microbial density was generally higher in biofilm exposed to 30 mg L^−1^ NO_3_^−^-N than in biofilm exposed to 5 mg L^−1^ NO_3_^−^-N for the same substrate (Fig. 1F), suggesting that high nitrate stimulated the growth of biofilm.

### Richness and diversity of microbial communities

**Figure 2:**
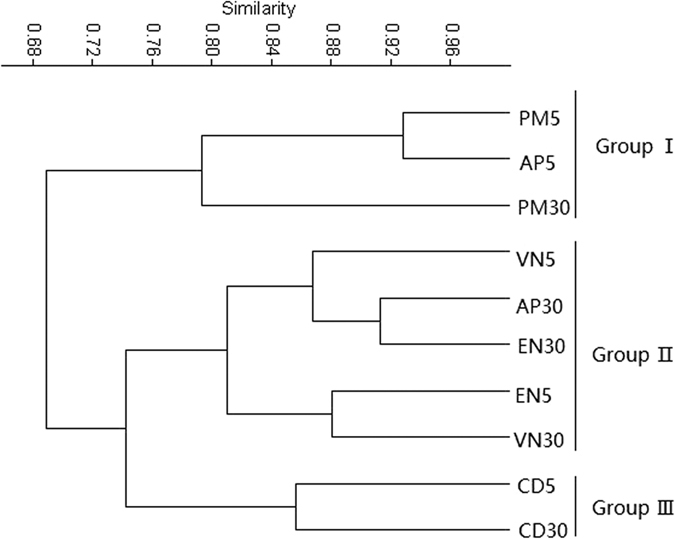
Cluster analyses of the bacterial community structure based on Bray-Curtis (5 and 30 indicate samples exposed to 5 and 30 mg L^−1^ NO_3_^−^-N, respectively). PM, *P*. *malaianus*; AP, artificial plants; VN, *V*. *natans;* EN, *E*. *nuttallii*; CD, *C*. *demersum*.

After filtering, high-quality reads of 16S rRNA genes ranging from 9088 (AP5) to 25982 (VN30) were obtained. The operational taxonomic units (OTUs) (at 3% cutoff) richness and Shannon values for the bacteria ranged from 758 (PM30) to 1579 (EN30) and 4.63 (PM30) to 6.33 (EN30) (Table S2), respectively, demonstrating that these substrates provided different niches for these microbes. Shannon values rapidly increased within 2000 reads of 16S rRNA genes and gradually reached stability thereafter (Figure S4). Good’s coverage of the ten samples ranged from 93.4% to 96.6%, indicating that the sequence libraries constructed in the current study covered most of the bacteria in these biofilms. Figure 2 shows that the bacterial communities in all 10 samples could be clustered into three groups based on OTU composition (OTUs occurring in fewer than three samples were removed). In general, the samples from the same type of bio-substrate were clustered into the same group.

**Figure 3:**
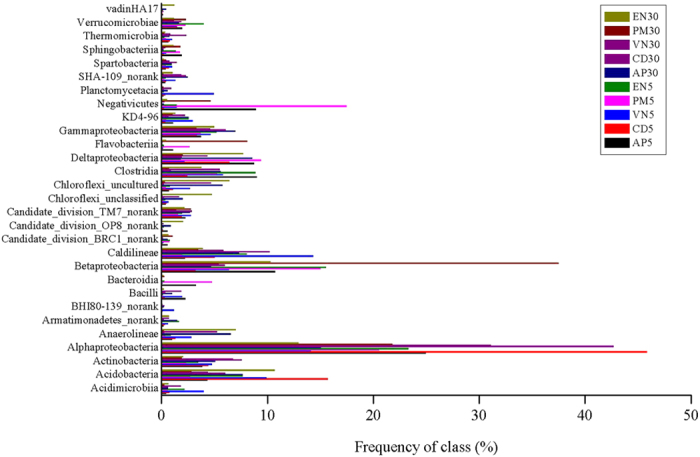
Distribution of the abundant bacterial classes (>1% frequency in at least one sample) in ten samples (5 and 30 indicate samples exposed to 5 and 30 mg L-1 NO3–N, respectively). PM, *P*. *malaianus*; AP, artificial plants; VN, *V*. *natans;* EN, *E*. *nuttallii*; CD, *C*. *demersum*.

Proteobacteria (accounting for 27.3–64.8% of the total effective bacterial sequences) was the most abundant phylum in all samples (Figure S5), followed by Chloroflexi, Firmicutes, Acidobacteria, Actinobacteria, Bacteroidetes, and Verrucomicrobia (Figure S5). For the same plant, the abundances of phylum Chloroflexi were obviously higher in biofilm exposed 30 mg L^−1^ NO_3_^−^-N than in that exposed to 5 mg L^−1^ NO_3_^−^-N, while the reverse trend was observed for the phylum Firmicutes. A total of 132 microbial classes were detected in ten samples, and 29 classes (>1%) dominated in at least one sample (Fig. 3 and Table S3). Within Proteobacteria, *α*-, *β*-, *δ*- and *γ*-Proteobacteria were observed in all ten biofilm samples. Class Acidobacteria, Actinobacteria, Candidate_division_TM7_norank, Caldilineae, and Verrucomicrobiae were predominant in all ten biofilm samples.

**Figure 4:**
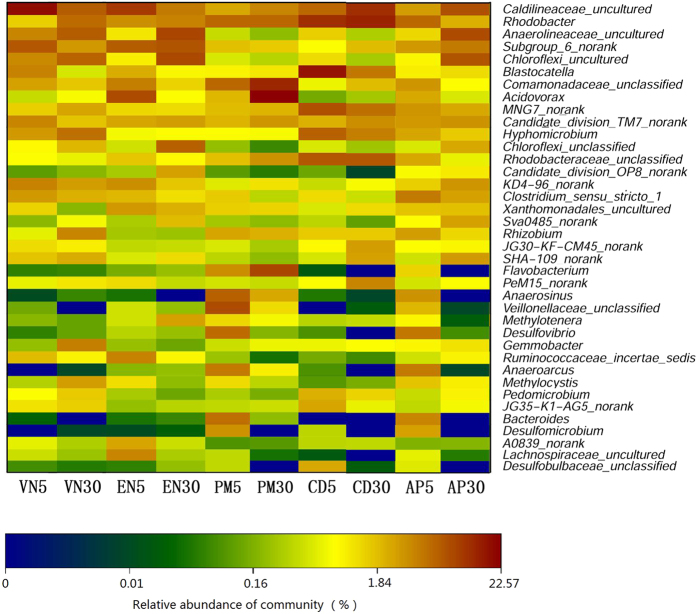
Heat map of the top 10 genera (%) in at least one sample (5 and 30 indicate samples exposed to 5 and 30 mg L^−1^ NO_3_^−^-N, respectively). PM, *P*. *malaianus*; AP, artificial plants; VN, *V*. *natans;* EN, *E*. *nuttallii*; CD, *C*. *demersum*.

A total of 38 genera belonging to the top 10 most abundant genera in at least one of samples were selected (Fig. 4). Among these top 10 abundant genera, five genera (*Rhodobacter*, *Caldilineaceae*_uncultured, *Candidate_division*_*TM*7_norank, *MNG*7_norank and Subgroup_6_norank) were distributed in at least six biofilm samples. The genus *Rhodobacter* occurred in 1.54–12.33% of the reads and was more abundant in samples exposed to 30 mg L^−1^ NO_3_^−^-N than in those exposed to 5 mg L^−1^ NO_3_^−^-N, except for the samples obtained from artificial plants.

Notably, the frequencies of *Anaerolineaceae*_uncultured, *Caldilineaceae*_uncultured, *Chloroflexi*_uncultured, Subgroup_6_norank, KD4-96_norank and SHA-109_norank in biofilms on artificial plants were higher (100%) in 30 mg L^−1^ NO_3_^−^-N than in 5 mg L^−1^ NO_3_^−^-N; however, the abundance of these bacteria varied among biofilm samples from submerged macrophytes. Several of the top 10 most abundant genera were only observed in one of the five substrates.

### Nitrogen response-related genera and OTUs

In the present study, three nitrifying bacteria genera, including *Nitrospina*, *Nitrospinaceae* and *Nitrospira*, were detected. *Nitrospina* was only detected in two samples (VN30 (0.10%) and AP30 (0.04%)). *Nitrospinaceae* and *Nitrospira* occurred in 0.01–1.39% and 0.07–1.78% of the total reads from ten samples, respectively. Denitrifiers, including genera *Acidovorax*, *Azospira*, *Bacillus*, *Dechloromonas*, *Desulfovibrio*, *Flavobacterium*, *Hyphomicrobium*, *Meganema*, *Rhizobium*, *Rhodobacter*, *Rhodoplanes*, and *Thiobacillus*, accounted for 7.48 to 43.69% of total reads in the present study (Table S4). The abundance of denitrifiers was higher in biofilms exposed to 30 mg L^−1^ NO_3_^−^-N than in those exposed to 5 mg L^−1^ NO_3_^−^-N, except for *Elodea nuttallii* and artificial plants.

**Figure 5:**
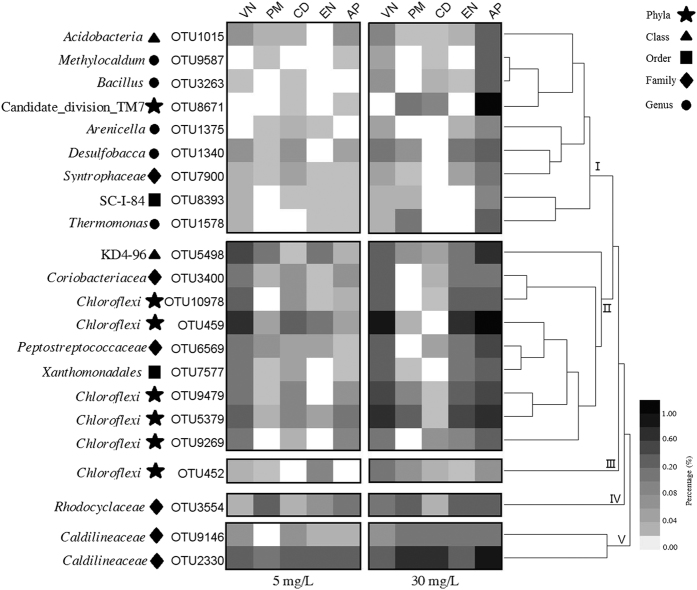
The 23 OTUs showing changes in abundance in response to nitrate nitrogen according to the redundancy analysis (RDA). PM, *P*. *malaianus*; AP, artificial plants; VN, *V*. *natans*; EN, *E*. *nuttallii*; CD, *C*. *demersum*.

A heat map generated using 23 OTUs of presumable denitrifier taxa showed changes in abundance in ten biofilm samples based on Redundancy analysis (RDA) (Fig. 5). The frequencies of these OTUs were higher in biofilms exposed to 30 mg L^−1^ NO_3_^−^-N than in those exposed to 5 mg L^−1^ NO_3_^−^-N. Several OTUs originated from the genera *Methylocaldum*, *Bacillus*, *Arenicella*, *Desulfobacca* and *Thermomonos*, whereas most of the sequences belonged to unclassified or uncultured bacteria at the genus level. Based on the cluster analysis (Fig. 5), five clusters represented nitrate-dependent changes.

**Figure 6:**
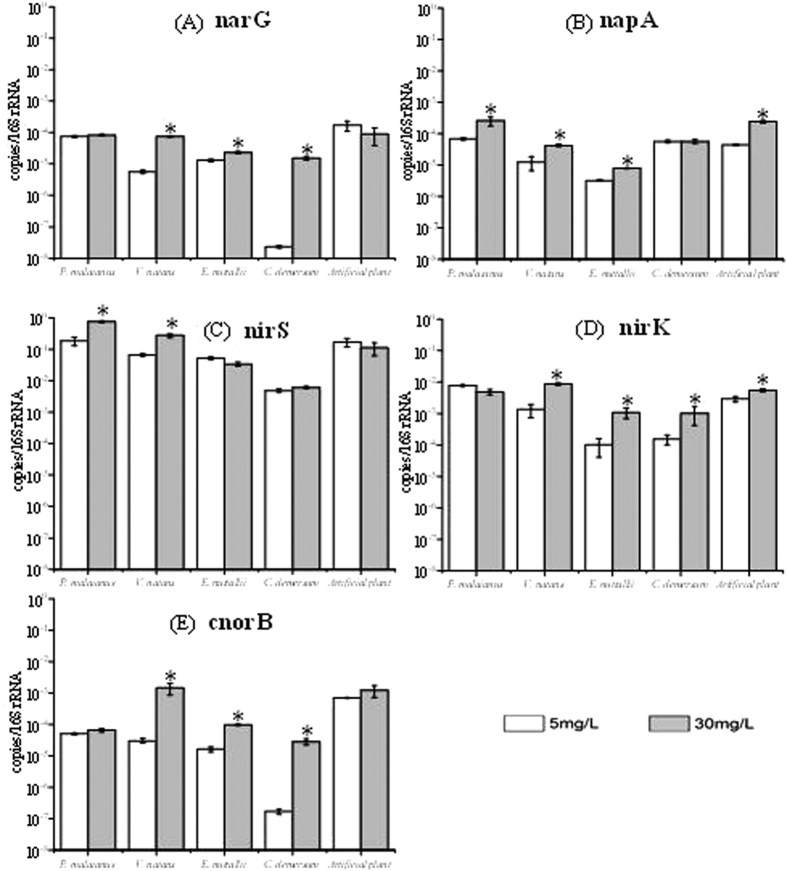
Relative abundance of five denitrification genes (**A**–**E**) in biofilms on five substrates at two nitrate concentrations. *Indicates significant differences at p < 0.05.

The relative abundances of five denitrifying genes (normalized to 16S rRNA genes) were measured using qPCR (Fig. 6). Compared with the abundance of genes in biofilm exposed to 5 mg L^−1^ NO_3_^−^-N, the abundance of *nar*G was significantly increased in biofilms from *V*. *natans*, *E*. *nuttallii* and *C*. *demersum*, while the abundance of *nap*A was increased in all biofilm samples except for biofilms from *C*. *demersum* exposed to 30 mg L^−1^ NO_3_^−^-N. Gene *nir*S was significantly increased in biofilms from the leaves of *P*. *malanius* and *V*. *natans*, and *nir*K was increased in biofilms from the leaves of *V*. *natans*, *E*. *nuttallii* and *C*. *demersum*. Upon nitrate treatments, the abundance of *cnor*B obviously increased in all biofilm samples. These data suggested that nitrate addition stimulated the bacteria carrying these genes.

### Responses of biofilm parameters and denitrifying gene abundances to environmental factors

**Figure 7:**
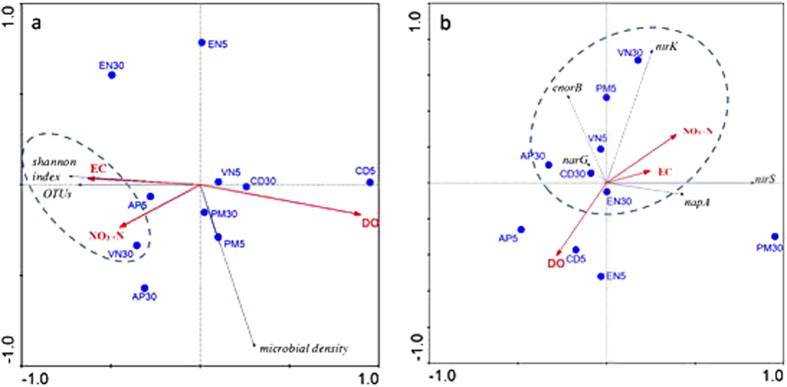
Redundancy analysis (RDA) map of the response of biofilm parameters (**a**) and denitrifying gene abundances (**b**) to environmental factors (5 and 30 indicate samples exposed to 5 and 30 mg L^−1^ NO_3_^−^-N, respectively). PM, *P*. *malaianus*; AP, artificial plants; VN, *V*. *natans;* EN, *E*. *nuttallii*; CD, *C*. *demersum*.

The impacts of environmental factors on biofilm parameters (Fig. 7a) and denitrifying gene abundance (Fig. 7b) were analyzed using a constrained redundancy analysis (RDA). The results showed that the Shannon index and OTU values (log transformed) were positively correlated with electrical conductivity and nitrate concentration; however, the microbial density was somewhat affected by dissolved oxygen (DO) (Fig. 7a). Five denitrifying genes, nitrate and electrical conductivity were primarily distributed in the same group (Fig. 7b).

## Discussion

Most natural microorganisms are not free in the growth environment but attach to solid surfaces in the form of biofilm. The bacteria attach to the media surface and other microbial communities through extracellular polymers, sticky substances secreted from bacteria, forming complex and stable filamentous or reticular structures.

Biofilm-like bacterial aggregates were also observed on the leaf surface of submerged macrophytes, and the biofilm cells were evenly distributed on artificial plants but appeared in microcolonies on the surfaces of submerged macrophytes. (Figs 1 and S2). Liang *et al*.^19^ observed that the number of microorganism species and the quantity and heterogeneity of emergent plant surface biofilms were all higher than those of gravel surface biofilms. We observed that the microbial densities varied on these substrates. The differences in microbial density might reflect the morphological characters and chemical composition of substrates, as many of these plants secrete allelopathically active plant exudates (such as polyphenols) that affect the epiphyte biomass^20^.

Aquatic macrophytes have been employed in wetlands for pollutant removal. However, the bacterial community structure on the submerged macrophytes in response to nitrogen remains unclear. A total of 11,834 OTUs were obtained from 130,639 sequences in the present study (Table S2), indicating that epiphytic biofilm is a species-rich micro-ecosystem. The results of the RDA analysis (Fig. 7a) revealed that the OTU numbers and Shannon index values were positively correlated with electrical conductivity and nitrate concentration, while microbial density was positively correlated with dissolved oxygen. Electrical conductivity strongly depends on the number of ions available to participate in conduction. Here, the electrical conductivity was used to determine the available nutrients derived from experimental systems and plant exudates. The results demonstrated that these nutrients determined the diversity of bacteria, while oxygen was one of the key factors determining the quantity of biofilms.

The results of the cluster analysis showed that compared with artificial plants, the bacterial community structure were somewhat host specific (Fig. 2). Recently, Li *et al*.^21^ reported that the diversity and richness of bacteria in planted beds was higher than that in unplanted beds. In addition, Liu *et al*.^22^ showed that the root systems of aquatic plants (emergent cattail, submerged hornwort, and floating *Lemna minor*) have selective effects on the microbial communities in wetlands, and wetlands with Cattail showed the highest community richness and diversity. These results suggested that the plant species obviously affected the bacterial community structure, as different species may produce specific exudates, specific leaching metabolites and proportions of chemicals that can impact the bacterial community structure^23^.

Consistent with these results (Table S3), Proteobacteria was the most abundant phylum on the leaf surfaces of aquatic angiosperms from Chesapeake Bay^24^. Proteobacteria, Bacteroidetes, Chloroflexi, Firmicutes, Actinobacteria and Verrucomicrobia were the most abundantly represented phyla on the leaves of lettuce^25^ and *Potamogeton crispus*^26^. Members of the Bacteroidetes phylum are abundant in aquatic habitats^27^. In phylum Proteobacteria, α-Proteobacteria was the most dominant class, except for sample PM30, where β-Proteobacteria was the most dominant class (Fig. 3 and Table S3). α-Proteobacteria was the most dominant group in biofilm from *Potamogeton crispus* in 12 freshwater lakes of China^26^, while β-Proteobacteria was the most abundant class on the leaves of two submerged macrophytes in Lake Taihu^28^. The percentages of unclassified or unknown species at the OTU level (Fig. 5) suggest that most of the bacteria in the natural environment cannot be cultured in an artificial medium^29^, and the diversity of the uncultured bacteria is considerable^30^.

Submerged macrophytes might play an important role in regulating the nutrient balance of water, reflecting the increased surface available to epiphytic microorganisms. However, few studies have examined the occurrence of nitrifiers and denitrifiers in epiphytic communities^31^. Three genera of nitrifiers were detected in the present study (Table S4). Members of nitrifiers have been found to be the dominant organisms in thermal artesian springs^32^ and in the sludge of wastewater treatment plants^33^. A recent study^34^ showed that the abundance of nitrifying bacteria typically represents less than 1% of the total bacterial population in activated sludge, although those microbes are extremely important for nitrogen removal. Denitrifiers, including the genera *Acidovorax*, *Azospira*, *Bacillus*, *Dechloromonas*, *Desulfovibrio*, *Flavobacterium*, *Hyphomicrobium*, *Meganema*, *Rhizobium*, *Rhodobacter*, *Rhodoplanes*, and *Thiobacillus*, play an important role in treating mature landfill leachate^35^ and wastewater^36^. For example, *Dechloromonas* oxidizes benzene using nitrate as an electron acceptor^37^. Species in the genus *Rhodobacter* (for example *Rhodobacter sphaeroides*) are photosynthetic bacteria, growing aerobically using photosynthetic electron transport or anaerobic respiration^38^, and some *Rhodobacter* species contribute substantially to denitrification^39^.

The microbial community in wetland environments is highly complex^40^, and most of the microorganisms cannot be cultivated. Therefore, the denitrifying genes were employed to characterize the denitrifier communities. All five denitrifying genes were detected in ten biofilm samples (Fig. 6). The genes *nar*G and *nap*A encode the subunits of two types of dissimilatory nitrate reductases Nar (membrane protein) and Nap (periplasmic binding protein), respectively^41^. NapA has almost exclusively been observed in the phylum Proteobacteria and can be expressed under fully aerobic conditions^9^. Nitrite is converted to NO or N_2_O through nitrite reductase (Nir), and *nir*K and *nir*S are the most widely used gene markers^41^. Genes encoding cnor proteins, which catalyze nitric oxide reduction at the outside of the cytoplasmic membrane, as well as the *cnor*B gene have been detected in aerobic denitrifiers^9^. Gaseous ^15^N losses via denitrification were significantly and positively associated with the abundance of *nir*K, *nir*S, and *nos*Z genes in eutrophic water^42^. The results of the present study showed that the abundances of denitrifying genes were positively correlated with nitrate (Fig. 7b). Taken together, these results demonstrated that nitrite application stimulated denitrifying microbes in biofilms.

Bacteria harboring different denitrifying genes might be amplified or attenuated under different environmental conditions and consequentially change the profiles of denitrifying genes in different environments^43^. DO might be one of the key parameters affecting the abundance of denitrifying genes^9^. The results of the RDA showed that the abundance of denitrifying genes was negatively correlated with DO (Fig. 7b). *Nap* is expressed under fully aerobic conditions and has been implicated in aerobic denitrification. *Nap*A has been used as a biomarker to evaluate the diversity of the nitrate-reducing microbial community^44^,^9^. The expression of *Nir* and *Nor* requires low oxygen^45^, and *Nir* expression in aerobic environments might be a common phenomenon^46^. In the present study, the DO concentrations ranged from 4 to 5.3 mg L^−1^ in water columns with 5 mg L^−1^ nitrate and from 2.9 to 4.9 mg L^−1^ in water columns with 30 mg L^−1^ NO_3_^−^-N in the daytime. However, the DO concentrations were generally lower at nighttime than those at daytime, reflecting the continuous respiration and periodic photosynthetic reactions in wetland systems. The low DO concentration might favor denitrification, as most denitrifiers most efficiently convert nitrate at a certain DO concentration, or optimal point, typically ranging from 3 to 5 mg L^−1^ for culture in sealed containers^9^. These data suggested that the environmental DO concentration in the present study was beneficial for aerobic denitrifiers in biofilms.

We concluded that the distribution of biofilms on the leaf surface of submerged macrophytes was different from that on artificial plants. Submerged macrophytes provided special niches for biofilms and determined the bacterial community in biofilms. The phyla Proteobacteria, Chloroflexi, Firmicutes, Acidobacteria, Actinobacteria, Bacteroidetes and Verrucomicrobia were predominant in these biofilms. The results of the RDA revealed that the OTUs numbers and Shannon index values were positively correlated with electrical conductivity and nitrate concentration, while microbial density was positively associated with dissolved oxygen. The abundances of denitrifying genes were positively correlated with nitrate but negatively correlated with DO. These results suggested that submerged macrophytes play an important role in determining the bacterial community structure, whereas nitrate stimulated the denitrifiers in biofilms. However, the mechanisms by which submerged macrophytes-biofilm systems remove nitrogen should be addressed in the future.

## Materials and Methods

### Experimental setup

Four submerged macrophytes with different morphologies were used in the present study (see supporting information Figure S6). The leaves of *Vallisneria natans* (VN) arise in clusters from the roots and have rounded tips and defined raised veins. The leaves of *Potamogeton malaianus* (PM) arise from the stems. The stem plants *Ceratophyllum demersum* (CD) and *Elodea nuttallii* (EN) have stiff whorls of needle-like leaves and thin branching stems with whorls of flat leaves at intervals, respectively. The single-leaf surface areas of four macrophytes were generally in the sequence VN > PM > EN > CD, whereas the reverse trend was observed for leaf numbers. Because the microbial community in biofilms on artificial plants is primarily modified through environmental factors compared with those on biotic substrates, artificial plants were used as controls to investigate the impact of plant types and nitrates on microbial community structure.

Artificial (polyvinyl chloride) plants and the plants of four submerged macrophytes were obtained from an aquatic plants company (Gaochun Futian Aquatic Macrophyte Company, Nanjing, China). After the old leaves were removed, healthy plants at similar growth stages were cultivated in 100 L tanks with 70 cm depth of water and 10 cm sediment. The sediment was obtained from Lake WulongTan, Nanjing, China. The plants occupied 50–70% of the water column. A total of 30 tanks were used, including three biological replicates.

After acclimation for seven days, the plants in three tanks were exposed to 5 mg L^−1^ NO_3_^−^-N, and the other three tanks were exposed to 35 mg L^−1^ NO_3_^−^-N for the same plants. The experiment was conducted in a greenhouse with a black 70% mesh shade to prevent intense light. The concentrations of nitrate were monitored daily, and the nitrate concentrations were maintained at the initial level to balance the nitrogen budget resulting from experimental systems using different plants. Based on preliminary experiments, nitrate was generated using a mixture (pH = 6.5) of CaNO_3_, NaNO_3_, KNO_3_ and HNO_3_ to avoid salt stress. The plant samples were collected at the end of the experiment (thirty days). DO and electrical conductivity were determined daily using a portable multi-parameter analyzer (HACH, USA). Total dissolved nitrogen and nitrate nitrogen were determined using an AA3 flow continuous chemistry analyzer (SEAL, Germany) according to the manufacturer’s instructions.

### Scanning electron microscopy (SEM)

After nitrate treatments for 30 days, middle-upper leaf samples (10–15 cm below water surface) were carefully collected from four submerged macrophytes and artificial plants and cut into small pieces. The small leaf samples were fixed with glutaraldehyde (2.5% in 50 mmol/L sodium cacodylate). Upon fixation, the samples were incubated in an osmium tetroxide solution (1% in 50 mmol/L sodium cacodylate buffer) in the dark. The fixed samples were dehydrated using a serial concentration of ethanol (20%, 40%, 60%, 70%, 80% and 90% ethanol) for 15 min at each concentration. After immersing twice in 100% ethanol for 15 min each, the samples were freeze dried. The dried samples were visualized using SEM (S3400, Hitachi, Japan) after sputter coating with gold.

### Biofilm sample collection and cell counting

Leaf samples from healthy plants (approximately 30 g) and artificial plants growing at 0–50 cm below the water surface were harvested to prevent contamination from sediments during harvesting. The harvested samples were transferred into a sterile 500 mL polyethylene bottle containing 400 mL of 50 mM phosphate-buffered saline (PBS, pH = 7.4) solution. Epiphytic microbes were detached after 3 min of ultra-sonication, 30 min of shaking (225 r/min) and subsequent ultra-sonication for 3 min^28^. The leaves were collected into a new tube containing 100 mL of PBS (pH 7.4) and treated with ultra-sonication for 3 min, followed by shaking at approximately 225 rpm for 30 min. The suspensions from the same sample were combined. For DNA isolation, the suspension samples were passed through a sieve with a mesh size of 50 μm to remove plant debris and subsequently centrifuged at 8000 rpm for 10 min. The pellets were collected and stored at −80 °C.

For cell counting, the microbes were collected using the above-mentioned methods without centrifugation. The suspension solution was combined for the same sample and fixed with 2% formaldehyde. The suspension samples (100 μL) were mixed with 700 μL of DAPI (10 μg mL^−1^) and incubated in the dark for 30 min. The samples were filtered through a 0.22 μm incubated black filter. The number of bacteria on the black membrane was counted under a fluorescence microscope (ZEISS, Germany). The surface area of leaves was calculated according to Leroy^47^.

### DNA extraction and RT-PCR analysis

Biofilm DNA was extracted using the Power Biofilm DNA Isolation kit (Mo Bio Laboratories, USA) according to the manufacturer’s instructions. The abundance of bacterial 16S rRNA, archaea bacteria 16S rRNA, periplasmic nitrate reductase (*nap*A) and membrane-bound nitrate reductase (*nar*G), nitrite reductase (*nir*S and *nir*K) and nitric oxide reductase (*qnor*B) genes were identified in the DNA samples from three biological replicates of each experiment set. Quantitative polymerase chain reaction (qPCR) using a MyiQ2 real-time PCR Detection System (Bio-Rad) with the fluorescent dye SYBR-Green approach was employed using the 16S rRNA gene and functional gene amplification. The amplification of qPCR was performed in 20 μL reaction mixtures containing 10 μL of SYBR Green I PCR master mix (Bio-Rad), 1 μL of template DNA (sample DNA or plasmid DNA for standard curves), 0.5 μL of forward and reverse primers, and 8 μL of sterile water. The qPCR protocol included 40 cycles. The primer sequences and qPCR protocols are provided in Table S1.

### 454 pyrosequencing

For *454 pyrosequencing*, three extracted biofilm DNA samples from one of three biological replicates were combined. The bacterial 16S rRNA gene was amplified using the primers 342F (5′-CTACGGGGGGCAGCAG-3′) and 806R (5′-GGACTACCGGGGTATCT-3′)^48^. The PCR reactions were performed in a 20 μL reaction mixture containing 4 μL of 5X FastPfu Buffer, 2 μL of 2.5 mM dNTPs, 0.8 μL of each primer (5 μM), 0.4 μL of FastPfu Polymerase, and 10 ng of template DNA. The amplification program included an initial denaturation step at 95 °C for 2 min, followed by 25 cycles at 95 °C for 30 s (denaturation), 55 °C for 30 s (annealing) and 72 °C for 30 s (extension), with a final extension at 72 °C for 5 min. Prior to sequencing, each PCR product was purified using the AxyPrep DNA Gel Extraction Kit (Axygen Biosciences, Union City, CA, U.S.) and quantified using QuantiFluor™ -ST (Promega, U.S.). Amplicons from different samples were subsequently mixed to achieve equal mass concentrations in the final mixture, which was subsequently used for pyrosequencing on a Roche 454 GS FLX+ Titanium platform (Roche 454 Life Sciences, Branford, CT, U.S.) according to the manufacturer’s instructions.

### Data processing and statistical analysis

The low-quality sequences with average quality scores <20 over a 50 bp sliding window and sequences shorter than 200 bp, with homopolymers longer than six nucleotides and containing ambiguous base calls or incorrect primer sequences were removed from the pyrosequencing-derived datasets. The high-quality sequences were assigned to samples according to barcodes. The sequences were aligned in accordance with SILVA alignment^49^ and cluster analysis at phylum level was performed based on the Bray–Curtis dissimilarity methods in the software PAST version 2.17. OTUs reaching 97% similarity level were used for diversity^50^, richness (Ace), Good’s coverage, and rarefaction curve analyses using Mothur^51^. The phylogenetic affiliation of each 16S rRNA gene sequence was analyzed using the RDP Classifier (http://rdp.cme.msu.edu/) against the SILVA (SSU 115) 16S rRNA database with a confidence threshold of 70%^52^. The 16S rRNA gene sequences derived from pyrosequencing have been deposited in the NCBI Sequence Read Archive under accession number PRJNA298644.

The data for the microbial densities were expressed as the means ± standard errors (n = 3) and analyzed using Student’s t test. A Bray-Curtis index of dissimilarity cluster analysis was conducted using the statistical software PAST version 3.10. The RDA was performed using CANOCO (Version 4.5) to explore the correlation between environmental parameters and the denitrifying gene distribution. The abundances of OTUs were log_10_ transformed for RDA.

## Additional Information

**How to cite this article**: Zhang, S. *et al*. Responses of bacterial community structure and denitrifying bacteria in biofilm to submerged macrophytes and nitrate. *Sci. Rep.* **6**, 36178; doi: 10.1038/srep36178 (2016).

**Publisher’s note:** Springer Nature remains neutral with regard to jurisdictional claims in published maps and institutional affiliations.

## Supplementary Material

Supplementary Information
