# Supplementary material for: Responses of bacterial community structure and denitrifying bacteria in biofilm to submerged macrophytes and nitrate
Source: Sci Rep. 2016 Oct 26;6:36178. doi: 10.1038/srep36178 (PMC5080643; doi:10.1038/srep36178)
Supplement: Supplementary Information [file srep36178-s1.pdf]

Response of bacterial community structure and denitrifying bacteria in biofilm to submerged macrophytes and nitrate

Songhe Zhang*[,a], Si Pang[a], Peifang Wang*[,a], Chao Wang[a], Chuan Guo[a], Felix Gyawu Addo[a], Yi Li[a].

[a]Ministry of Education Key Laboratory of Integrated Regulation and Resource Development on Shallow Lakes, College of Environment, Hohai University, Nanjing 210098, China

*corresponding author, PFWang2005@hhu.edu.cn; shzhang@hhu.edu.cn

RESULTS AND DISCUSSION

During the experimental processes, the level of dissolved oxygen and electronic conductivity did not alter significantly in each treatment **(Figure S1)**. For the same plants, the electronic conductivity was generally lower in 5 mg L^{-1} than in 30 mg L^{-1}. The nitrogen removal potential ranged from 1.2 (AP5) to 2.6 (AD30) mg NO_3^{-}N m^{-3} day^{-1} in water column and was obviously higher in submersed macrophytes dominant systems (mean values 2.22) than artificial plants (mean values 1.42). These data demonstrated that the plant-biofilm systems contributed to the nitrate removal.

As revealed by 454 pyrosequencing (Table S2), the occurrence of Archaeal OTUs ranged from 0.02% (PM30) to 0.23% (AP30) of total reads from each sample. Base on the RT-PCR results, the relative abundances of archaebacteria (ARC, Figure S3) ranged from 0.1% (CD5) to 4.15% (AP30). These results suggested that primers archaebacterial existed in the biofilm but occurred in low frequencies.

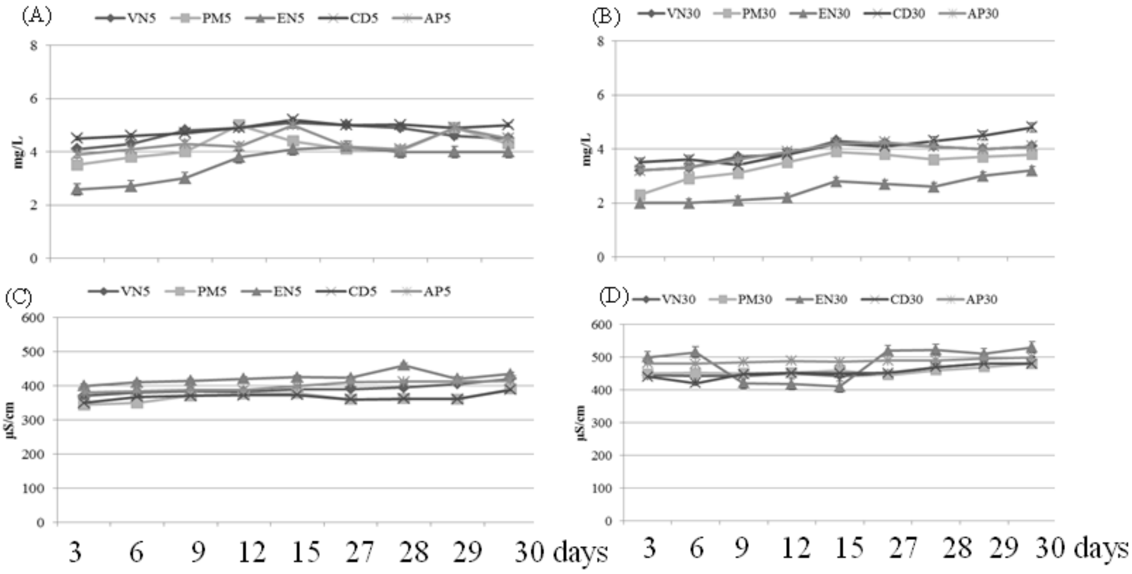

Figure S1 Dissolved oxygen (A and B) and electrical conductivity in water column at intimal experiment (3-15 days) and end of experiment (27-30 days) (5 and 30 indicates samples exposed to 5 and 30 mg L^{-1} NO_3–N, respectively, PM, *P. malaianus*; AP, artificial plants; VN, *V. natans;* EN, *E. nuttallii*; CD, *C. demersum.*)..

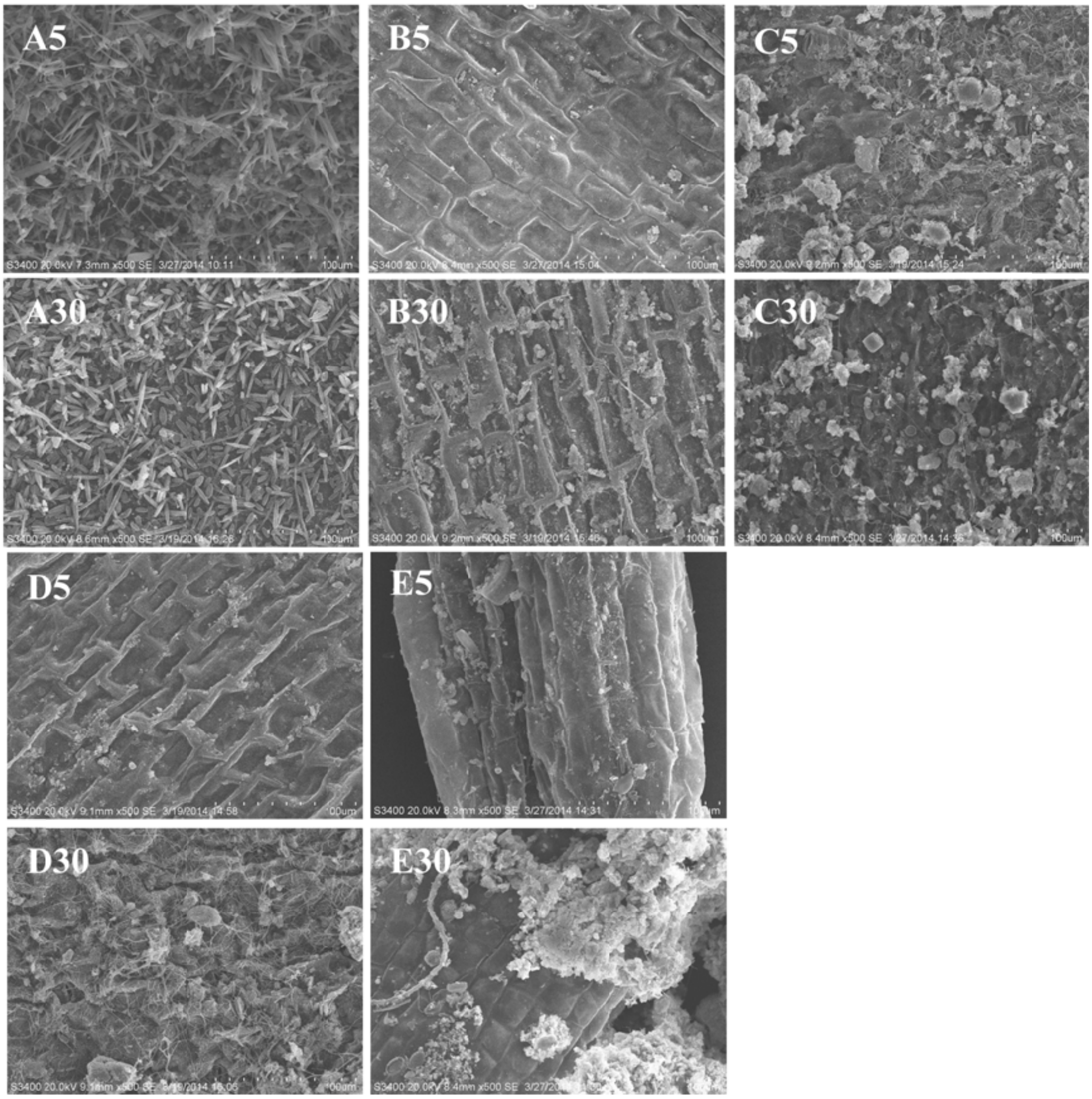

Figure S2 SEM micrographs of biofilm on submerged macrophytes under 100μm. (A5) artificial plants; (B) *Elodea nuttallii*; (C) *Potamogeton malaianus*; (D) *Vallisneria natans;* (E) *Ceratophullum* (5 and 30 indicates samples exposed to 5 and 30 mg L^{-1} NO_3–N, respectively).

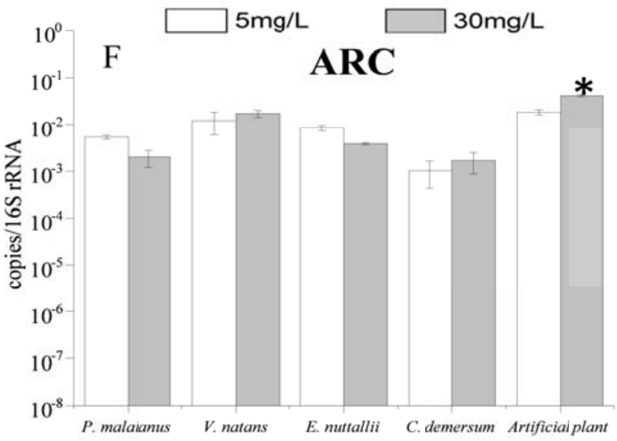

Figure S3 The density of attached microorganism in biofilm exposed to 5 and 30 mg L^{-1} NO_3^--N.

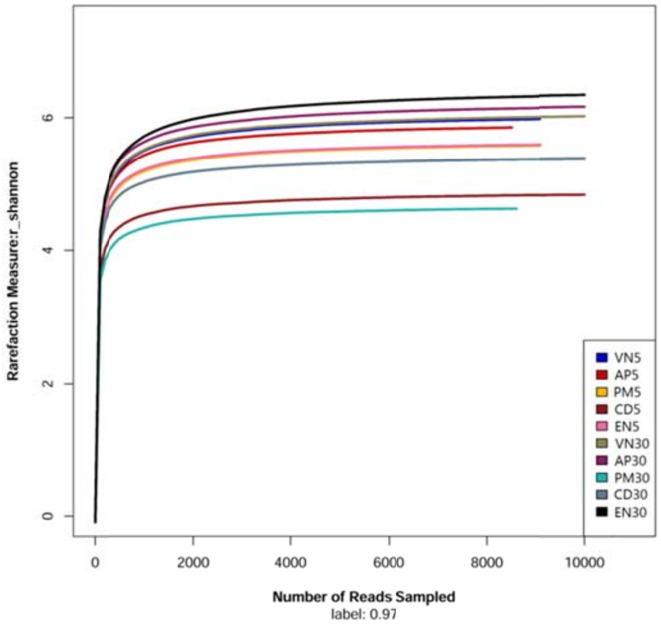

Figure S4 Shannon-Wiener curves base on pyrosequencing of bacterial communities in biofilms. 5 and 30 indicates samples exposed to 5 and 30 mg L^{-1} NO_3^--N, respectively; PM, *P. malaianus*; AP, artificial plants; VN, *V. natans;* EN, *E. nuttallii*; CD, *C. demersum.*

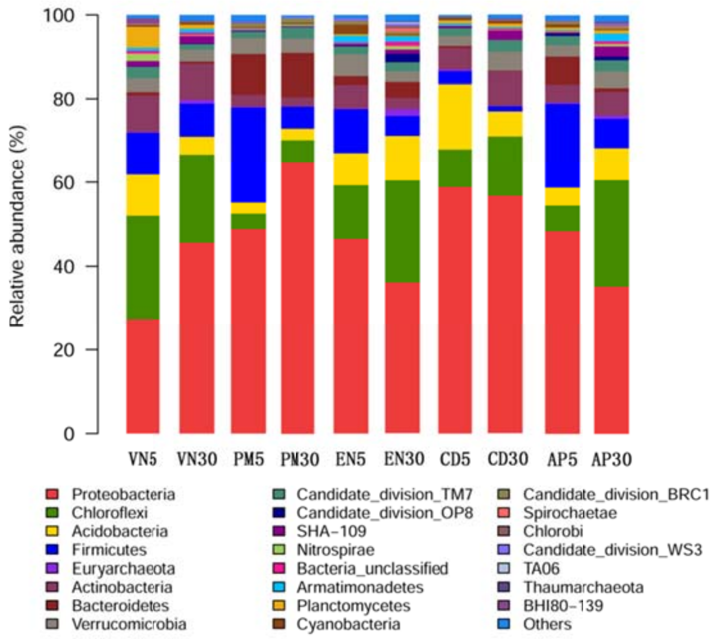

Figure S5 Relative abundances (%) phyla in the 10 biofilm samples including percentages of phyla below 1% in one sample. 5 and 30 indicates samples exposed to 5 and 30 mg L^{-1} NO_3-N, respectively; PM, *P. malaianus*; AP, artificial plants; VN, *V. natans;* EN, *E. nuttallii*; CD, *C. demersum.*

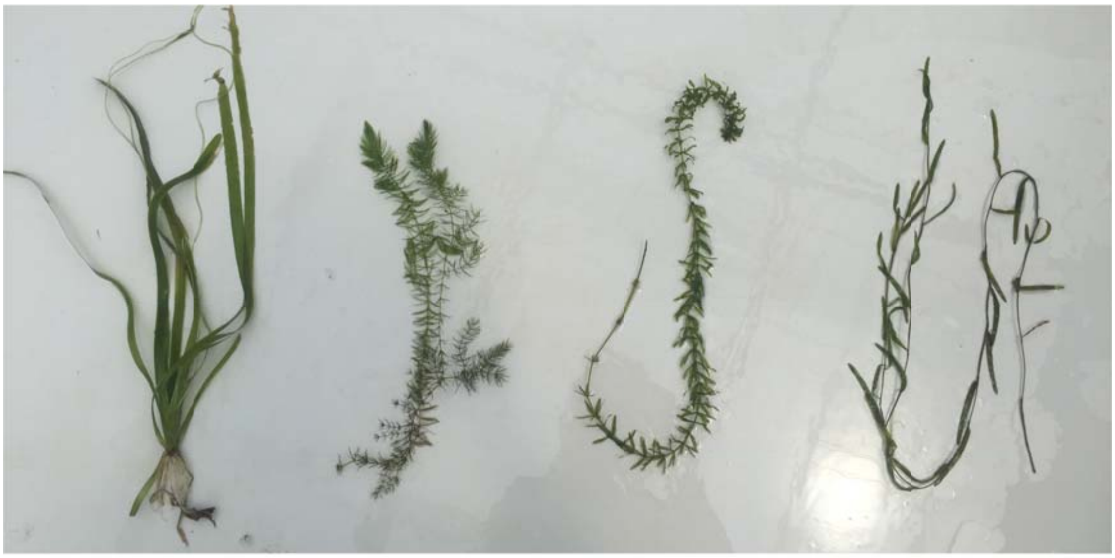

Vallisneria natans   *Ceratophyllum demersum*   *Elodea nuttallii*   *Potamogeton malaianus*

Figure S6 Characterizations of *four* submersed macrophytes

Table S1 The sequence of primers used in this study.

Primer	Sequence (5'-3')	Reference
Arch-amoAF	STAATGGTCTGGCTTAGACG	1
Arch-amoAR	GCGGCCATCCATCTGTATGT	
NirS cd3F	GTSAACGTSAAGGARACSGG	2
NirS 3cdR	GASTTCGGRTGSGTCTTGA	
nirK 1F	GGMATGGTKCCSTGGCA	3
nirK 5R	GCCTCGATCAGRTTRTGGTT	
cnorB-2F	GACAAGNNNTACTGGTGGT	4
cnorB-6R	GAANCCCCANACNCCNGC	
narGT37	CAYGGNGTNAAYTGYACNGG	5
narG W9	MGNGGNTGYCCNMGNGGNGC	
NapAV16	GCNCCNTGYMGNTTYTGYGG	6
NapAV66	DATNGGRTGCATYTCNGCCATRT	

Table S2 Characterization and analysis of 16S rRNA gene reads.

Sample name	Reads			3% cutoff			
	Valid	Trimed	Trimed vs Valid (%)	OTUs		Shannon value	Good`s coverage (%)
				Archaea	Bacteria		
AP5	10184	9088	89.2	11(0.12%*)	1137	5.85	94.6
AP30	11325	10635	93.9	24(0.23%*)	1471	6.16	93.8
EN5	11353	10318	90.9	8(0.08%*)	1045	5.6	95.3
EN30	12112	11435	94.4	44(0.38%*)	1579	6.33	93.4
VN5	11092	10017	90.3	11(0.11%*)	1329	5.98	93.7
VN30	28449	25982	91.3	24(0.09%*)	1434	6.02	93.9
PM5	12484	10038	80.4	9(0.09%*)	1044	5.58	95.5
PM30	10525	9092	86.4	2(0.02%*)	758	4.63	96.6
CD5	23017	21573	93.7	15(0.07%*)	896	4.84	96
CD30	13075	12461	95.3	5(0.04%*)	987	5.39	96.2

*indicates the percentages of archaeal reads in total reads.

5 and 30 in sample name indicate samples exposed to 5 and 30 mg L^{-1} NO_3^--N, respectively.

PM, *P. malaianus*; AP, artificial plants; VN, *V. natans;* EN, *E. nuttallii*; CD, *C. demersum.*

Table S3 Abundances of top10 class (bold figures) in at least one sample (%).

Phylum	Class	VN5	VN30	PM5	PM30	CD5	CD30	EN5	EN30	AP5	AP30
Acidimicrobiia	Acidimicrobiia	**3.96**	1.82	0.54	0.26	0.7	0.61	2.15	0.65	0.42	0.6
Acidobacteria	Acidobacteria	**9.91**	**4.35**	**2.68**	**2.78**	**15.66**	**6.01**	**7.66**	**10.67**	**4.31**	**7.65**
Actinobacteria	Actinobacteria	**4.76**	**6.73**	**2.28**	1.9	**4.39**	**7.54**	**3.44**	2	**3.84**	**5.06**
Bacteroidetes	Bacteroidia	0.09	0.01	**4.75**	0.24	0.02	0	0.25	0.16	**3.21**	0.03
	Flavobacteriia	0.1	0.06	**2.63**	**8.09**	0.07	0	0.19	0.43	1.06	0.09
Candidate_division_TM7	Candidate_division_TM7_norank	2.75	1.35	1.55	**2.76**	**1.87**	**2.83**	1.93	2.16	**2.24**	2.68
Chloroflexi	Anaerolineae	2.81	**5.22**	0.33	0.21	1.28	0.25	0.86	**6.97**	1	**6.52**
	Caldilineae	**14.3**	**5.81**	1.92	**3.44**	**4.98**	**10.17**	**8.01**	**3.86**	2.19	**7.29**
	Chloroflexi_unclassified	0.65	1.61	0.15	0.06	0.44	0.2	0.32	**4.73**	0.36	1.99
	Chloroflexi_uncultured	2.66	**4.65**	0.37	0.28	1.08	0.22	0.76	**6.37**	0.66	**5.74**
	KD4-96	**2.91**	2.2	0.41	1.04	0.34	0.63	**2.55**	1.27	1.08	2.52
	Thermomicrobia	0.98	0.79	0.4	0.26	0.75	**2.31**	0.26	0.31	0.65	0.73
Cyanobacteria	Cyanobacteria	0.44	0.56	0.27	0.37	0.67	0.7	**2.3**	0.32	0.73	0.25
Firmicutes	Clostridia	**5.79**	**5.49**	**5.23**	0.67	**2.41**	0.83	**8.85**	**3.74**	**8.96**	**5.58**
	Negativicutes	1.42	0.23	**17.43**	**4.6**	0.2	0.04	1.42	0.52	**8.91**	0.22
Planctomycetes	Planctomycetacia	**4.93**	0.9	0.07	0.14	0.33	0.22	0.22	0.05	0.15	0.55
Proteobacteria	Alphaproteobacteria	**14.08**	**31.09**	**20.51**	**21.78**	**45.82**	**42.66**	**23.3**	**12.91**	**24.93**	**15.01**
	Betaproteobacteria	**6.33**	**5.39**	**15**	**37.46**	**3.17**	**5.94**	**15.49**	**10.28**	**10.71**	**4.67**
	Deltaproteobacteria	2.14	**4.32**	**9.39**	**1.97**	**6.37**	**1.89**	1.78	**7.68**	**8.74**	**8.56**
	Gammaproteobacteria	**4.62**	**4.59**	**3.66**	**3.29**	**3.3**	**6.06**	**5.19**	**4.97**	**3.68**	**6.93**
SHA-109	SHA-109_norank	1.28	1.83	0.25	0.17	0.42	**2.27**	0.37	1.01	0.33	2.45
Verrucomicrobia	Verrucomicrobiae	1.4	1.16	2.25	**2.29**	**1.46**	1.83	**3.96**	1.15	1.92	1.64

Table S4 The abundance of bacteria associated with nitrogen cycling in each sample (%).

	**Genus**	VN5	VN30	PM5	PM30	CD5	CD30	EN5	EN30	AP5	AP30
Nitrifying bacteria	*Nitrospina*	0	0.13	0	0	0	0	0	0	0	0.05
	Nitrospira	2.50	0.29	0.11	0.08	0.19	0.21	0.11	1.16	0.33	0.72
	Nitrospinaceae_uncultured	0.09	0.16	0.35	0.07	0.05	0.01	0.14	1.39	0.36	0.78
	**Sub-total**	**2.59**	**0.58**	**0.46**	**0.15**	**0.24**	**0.22**	**0.25**	**2.55**	**0.69**	**1.55**
Denitrifying bacteria	*Acidovorax*	0.40	0.79	2.12	22.57	0.15	0.26	8.09	0.87	2.50	0.51
	Azospira	0.09	0.33	0.59	1.81	0.15	0.06	1.22	0.43	0.38	0
	Bacillus	2.01	1.76	0.06	0.01	0.18	0.39	0.11	0.16	0.26	0.91
	Dechloromonas	0.20	0.49	0.79	0.47	0.19	0.03	0.21	0.69	0.46	0.53
	Desulfovibrio	0.06	0.12	5.50	0.18	0.08	0	0.34	0.23	4.68	0.08
	Flavobacterium	0.06	0.07	3.56	9.30	0.02	0	0.15	0.23	1.44	0
	Hyphomicrobium	3.06	5.33	0.88	0.79	6.34	4.26	0.75	0.87	2.60	1.67
	Meganema	0.37	0.55	0.23	0.31	0.41	1.09	0.21	0.59	0.31	0.12
	Rhizobium	0.62	3.90	2.62	2.34	1.66	1.59	0.26	0.24	2.98	1.38
	Rhodobacter	1.54	5.56	5.83	5.88	12.33	14.01	3.54	4.05	5.71	2.28
	Thiobacillus	0.19	0.07	0.14	0.03	0.02	0.01	0.06	0.32	0.02	0
	**Sub-total**	**8.6**	**18.97**	**22.32**	**43.69**	**21.53**	**21.7**	**14.94**	**8.68**	**21.34**	**7.48**

REFERENCES

1. Kowalchuk, G. A.; Stephen, J. R.; DeBoer, W.; Prosser, J. I.; Embley, T. M.; Woldendorp, J. W., Analysis of ammonia-oxidizing bacteria of the beta subdivision of the class Proteobacteria in coastal sand dunes by denaturing gradient gel electrophoresis and sequencing of PCR-amplified 16S ribosomal DNA fragments. *Applied and Environmental Microbiology* **1997,** *63* (4), 1489-1497.
2. Throback, I. N.; Enwall, K.; Jarvis, A.; Hallin, S., Reassessing PCR primers targeting nirS, nirK and nosZ genes for community surveys of denitrifying bacteria with DGGE. *Fems Microbiology Ecology* **2004,** *49* (3), 401-417.
3. Braker, G.; Fesefeldt, A.; Witzel, K. P., Development of PCR primer systems for amplification of nitrite reductase genes (nirK and nirS) to detect denitrifying bacteria in environmental samples. *Applied and Environmental Microbiology* **1998,** *64* (10), 3769-3775.
4. Braker, G.; Tiedje, J. M., Nitric oxide reductase (norB) genes from pure cultures and environmental samples. *Applied and Environmental Microbiology* **2003,** *69* (6), 3476-3483.
5. Gregory, L. G.; Karakas-Sen, A.; Richardson, D. J.; Spiro, S., Detection of genes for membrane-bound nitrate reductase in nitrate-respiring bacteria and in community DNA. *Fems Microbiology Letters* **2000,** *183* (2), 275-279.
6. Flanagan, D. A.; Gregory, L. G.; Carter, J. P.; Karakas-Sen, A.; Richardson, D. J.; Spiro, S., Detection of genes for periplasmic nitrate reductase in nitrate respiring bacteria and in community DNA. *Fems Microbiology Letters* **1999,** *177* (2), 263-270.
